# That Wave of Prosperity

**Published:** 1897-10

**Authors:** 


### THAT WAVE OF PROSPERITY.

The politicians have been predicting to us for many months the advent of a wave of prosperity, and their opponents have hooted at them and reviled them in the various modes which came to hand, and were most convenient. That the wave is upon us we have not the least particle of doubt, but that politics had anything to do with it we know is not true.

The shortage of Europe on wheat and our phenomenal crops in the cereal have certainly to be awarded the credit of the wave of prosperity which has begun to manifest itself in the commercial world. We are exchanging our golden grain for the golden coin of Europe, and had we more of the products of the earth they would be eagerly bought up by our trans-Atlantic friends. The American farmer is beginning to realize upon his labor, and he has the prospect before him of being able to indulge in more than the absolute necessities of life. He has, at least, an opportunity of being able to assert his manhood in the way, among others, of paying his debts like a man. Not the least among his creditors is his physician who, at all times, and in all sorts of weather, has attended to the physical ills of himself and his dear ones. The country practitioner is certainly among those who is destined to profit by the wave of prosperity upon us, and no one more deservedly so. His life, usually so full of hardship, privation, and work, is destined to be made a little more pleasant by a substantial recognition of his services, and it will be tendered to him with an ungrudging hand. We are glad to know this, for if there be a generous, self-sacrificing and hard-working member of society it certainly is the country doctor. There are no roads too bad, no weather too unpropitious, to deter him from going on his work of mercy, often without any hope of reward, and, at best, with the prospect of but a miserable pittance in the event of times getting better. It is for this reason that we rejoice at the advent of the wave of prosperity. Our brethren located in the country, more especially in the West, will reap a substantial reward, and, at least, have some little encouragement to pursue their self-sacrificing task of ministering to the diseases of others.

It is a little encouragement of this sort that makes the country practitioner pluck up courage and continue to pursue the practice of his profession with renewed vigor. It also, in no small degree, exerts its influence upon the urban doctor, who no longer is asked to "book" everything, but sees some substantial acknowledgment of his services. May the wave which is coming continue its beneficent influence.—*St. Louis Medical and Surgical Journal.*

